# Safety and efficacy of intracardiac echocardiography-guided zero-fluoroscopy ablation in atrial fibrillation patients: a comparative study of high-power short-duration and low-power long-duration strategies

**DOI:** 10.3389/fcvm.2024.1510889

**Published:** 2024-11-21

**Authors:** Guang-an Liu, Bo Shao, Wanglong Wu, Linxiao Zhou, Jing Cui, Wenxue Chen, Ruoxi Zhang, Feng Liu

**Affiliations:** Department of Cardiology, Suzhou Kowloon Hospital, Shanghai Jiao Tong University School of Medicine, Suzhou, China

**Keywords:** zero-fluoroscopy, intracardiac echocardiography, high-power shortduration, atrial fibrillation, radiofrequency ablation

## Abstract

**Introduction:**

In atrial fibrillation (AF) ablation, fluoroscopy has been a standard tool for catheter guidance. However, the combination of electroanatomic mapping systems (EAMs) and intracardiac echocardiography (ICE) now allows for minimal or zero-fluoroscopy procedures. Concurrently, high-power short-duration (HPSD) ablation has emerged as a promising technique, offering enhanced resistive heating while reducing conductive heating. This approach potentially improves both safety and efficacy. Despite these advancements, there is a lack of comprehensive clinical data on the safety and effectiveness of HPSD ablation when used in conjunction with ICE-guided zero-fluoroscopy procedures.

**Objective:**

To compare two different ablation strategies—high-power short-duration (HPSD) and low-power long-duration (LPLD)—both utilizing intracardiac echocardiography (ICE)-guided zero-fluoroscopy in the context of atrial fibrillation (AF) ablation.

**Methods:**

This retrospective study included 173 consecutive patients with AF who underwent ICE-guided zero-fluoroscopy ablation. Patients were divided into two groups: HPSD and LPLD. All procedures were conducted using an EAM system with ICE guidance. Both groups underwent routine pulmonary vein isolation (PVI), with additional linear ablations performed for persistent AF when necessary. We compared treatment outcomes and the incidence of complications between the two groups.

**Results:**

All procedures were successfully completed under ICE-guided zero-fluoroscopy, establishing a feasible and reliable workflow. The procedure and ablation times were significantly shorter in the HPSD group compared to the LPLD group. At one-year follow-up, sinus rhythm was maintained in 77 patients in the HPSD group and 74 patients in the LPLD group, with no significant difference between the two group. Postoperative complications occurred in 5 patients in the HPSD group and 3 patients in the LPLD group. Importantly, there were no major adverse cardiac and cerebrovascular events (MACCE) in either group.

**Conclusion:**

A zero-fluoroscopy workflow utilizing an EAM system combined with ICE appears to be both feasible and safe for ablation in AF patients. In patients undergoing ICE-guided zero-fluoroscopy ablation, the HPSD strategy is comparable to LPLD ablation in effectiveness while offering the benefit of shorter procedure and ablation times.

## Graphical Abstract

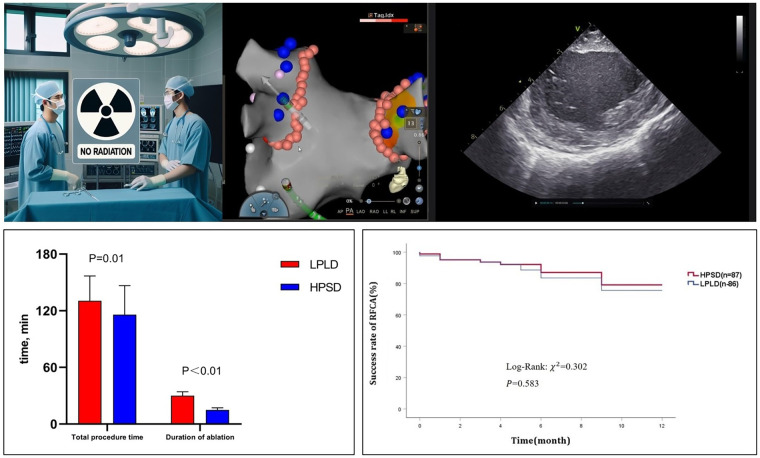

## 1 Introduction

For patients with symptomatic atrial fibrillation (AF) that is resistant to medication, radiofrequency catheter ablation (RFCA) has emerged as a primary treatment option. The foundation of AF ablation procedures typically involves pulmonary vein isolation (PVI) as the primary strategy for atrial fibrillation (AF) ablation, which can be achieved using various energy forms, including radiofrequency (RF), cryoablation, and pulsed field ablation (1, 2). Catheter ablation aims to produce continuous, transmural, and permanent lesions without harming nearby tissues or structures. The conventional approach, known as low-power long-duration (LPLD) ablation, generally employs power settings of 30–35 W. This method has demonstrated variable success rates, with one-year outcomes ranging from 59% to 89% effectiveness in maintaining normal heart rhythm post-procedure (1). However, pulmonary vein conduction recovery is a common issue (3). To enhance the success rate of RFCA, recent studies have investigated the efficacy and safety of the high-power short-duration (HPSD) ablation strategy. HPSD aims to maximize resistive heating while minimizing conductive heating, potentially offering a safer and more effective alternative (4–6).

Fluoroscopy has traditionally been a primary imaging tool during these procedures. In recent years, growing concerns have emerged regarding the cumulative effects of ionizing radiation exposure and musculoskeletal problems associated with wearing lead aprons during procedures. This has led to the development of techniques aimed at reducing fluoroscopy usage, aligning with the As Low As Reasonably Achievable (ALARA) principle (7). Recent technological progress in electroanatomic mapping systems (EAM) and the integration of intracardiac echocardiography (ICE) have revolutionized ablation procedures. These advancements now allow for the safe execution of such interventions with significantly reduced radiation exposure. This evolution in technique not only enhances patient safety but also provides improved protection for medical personnel involved in these procedures (8).

There have been few reports on completely zero-fluoroscopy RFCA procedures for AF. At our center, we have implemented ICE imaging guidance combined with EAM reconstruction to achieve a zero-fluoroscopy workflow. We reconstructed anatomical models of the right atrium and atrial septum using EAM, and employed ICE combined with EAM guidance for transseptal puncture (TSP), as well as left atrial reconstruction and ablation processes, gradually developing a completely zero-fluoroscopy catheter ablation technique. This study aimed to compare two different ablation strategies—HPSD and LPLD—both utilizing ICE-guided zero-fluoroscopy in the context of AF ablation.

## 2 Methods

### 2.1 Ethical statement

This study protocol conforms to the ethical guidelines of the 1975 Declaration of Helsinki, as reflected in *a priori* approval from the institution's human research committee. This study was also approved by the Research Ethics Committee of Suzhou Kowloon Hospital, Shanghai Jiao Tong University School of Medicine, China. The ethical approval number is HG-2024-013. Informed consent was obtained from all patients.

### 2.2 Study population

We conducted a study at Suzhou Kowloon Hospital, an affiliate of Shanghai Jiao Tong University School of Medicine, screening a total of 286 patients with non-valvular AF. Among them, 173 patients who underwent ICE-guided zero-fluoroscopy ablation between January 2019 and December 2022 were ultimately included in the study. The study included adult patients aged 18 and above who had been diagnosed with either paroxysmal or persistent AF and had shown resistance or intolerance to anti-arrhythmic drug (AAD) therapy. In accordance with the 2020 European Society of Cardiology (ESC) Guidelines for the Management of Atrial Fibrillation, which were developed in collaboration with the European Association for Cardio-Thoracic Surgery (EACTS) (1), paroxysmal AF is defined as an arrhythmia that resolves spontaneously or through cardioversion within a 7-day period (9).

In our study, we classified AF as persistent when the arrhythmia continued uninterrupted for a minimum of 7 days. We applied several exclusion criteria to ensure a focused and appropriate patient cohort. Individuals who had previously undergone AF ablation procedures were not eligible for participation. Additionally, we excluded patients with long-standing persistent AF, defined as an uninterrupted arrhythmic episode lasting more than one year. Safety considerations were paramount in our selection process. Consequently, we did not include patients with contraindications to anticoagulation therapy. Furthermore, the presence of intracardiac thrombus, as detected during pre-procedural screening, was grounds for exclusion due to the associated risks during ablation (9).

### 2.3 Preoperative preparation

In preparation for the procedure, all study participants underwent a minimum of one month of oral anticoagulation therapy. For patients on vitamin K antagonists (VKAs), the oral anticoagulant was discontinued 3 days before the procedure, and the INR was checked on the day of the procedure. If the INR was less than 1.5, 100 units/kg of unfractionated heparin was administered during the procedure. For patients taking new-oral-anticoagulants (NOAC), the last dose was taken the day before the procedure, with 100 units/kg of unfractionated heparin also administered during the procedure. ACT was routinely monitored during the procedure to maintain a range of 250–350 s. The ablation procedures were performed under local anesthesia. Anti-arrhythmic drugs (AADs) were discontinued at least 7 days before the scheduled intervention. To ensure patient safety, we conducted pre-procedural screening for intracardiac thrombus using either computed tomography (CT) or transesophageal echocardiography (TEE). During the ablation, unfractionated heparin was administered immediately following transeptal puncture. The dosage was adjusted as needed to maintain an activated clotting time exceeding 300 s throughout the procedure. It's worth noting that we did not employ an esophageal probe during these interventions.

### 2.4 Zero-fluoroscopy workflow

**Figure 1:**
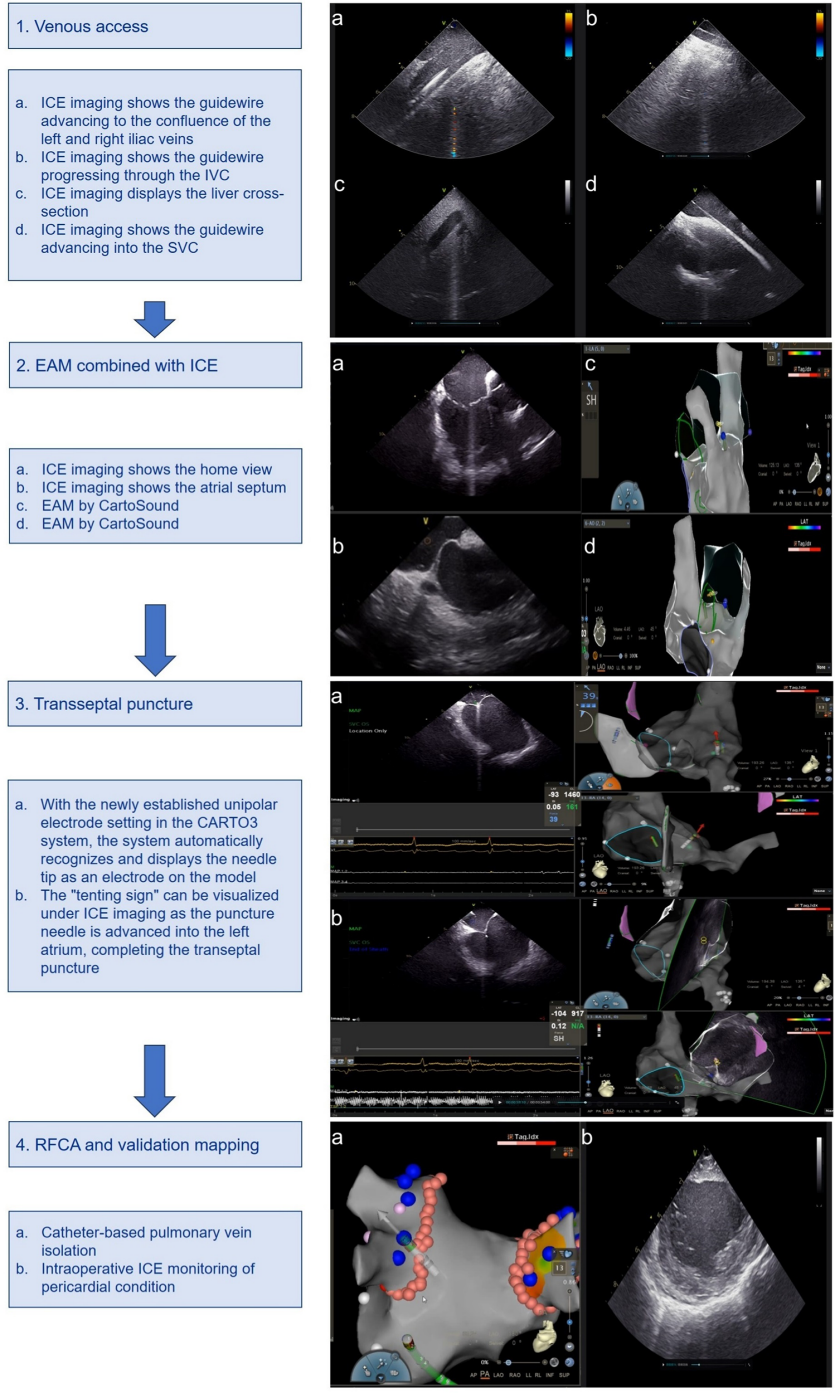
Zero-fluoroscopy workflow using ICE combined with EAM. ICE, intracardiac ultrasound; EAM, electroanatomic mapping; IVC, inferior vena cava; SVC, superior vena cava.

The workflow is described in the following steps: (1) venous access, (2) EAM combined with ICE, (3) transseptal puncture, (4) RFCA and validation mapping (Figure 1) (10).

Our procedure begins with the insertion of an ICE catheter via the left femoral vein. Using ultrasound imaging, we navigate this catheter into the right atrium. Subsequently, we utilize CartoSound technology to construct detailed anatomical models of key cardiac structures. These include the tricuspid valve, the ostium of the coronary sinus ostium (CSO), the aortic valve, the left atrial appendage (LAA), both left and right pulmonary veins, and the posterior wall of the left atrium.

The next phase involves accessing the right femoral vein, where we introduce 7F and 8F venous sheaths separately. The 8F sheath is then carefully advanced into the superior vena cava (SVC) with the aid of a guidewire. This meticulous approach to vascular access and cardiac mapping forms the foundation of our zero-fluoroscopy ablation technique, allowing for precise navigation and intervention without reliance on traditional fluoroscopic guidance (Figures 1-1).

**Figure 2:**
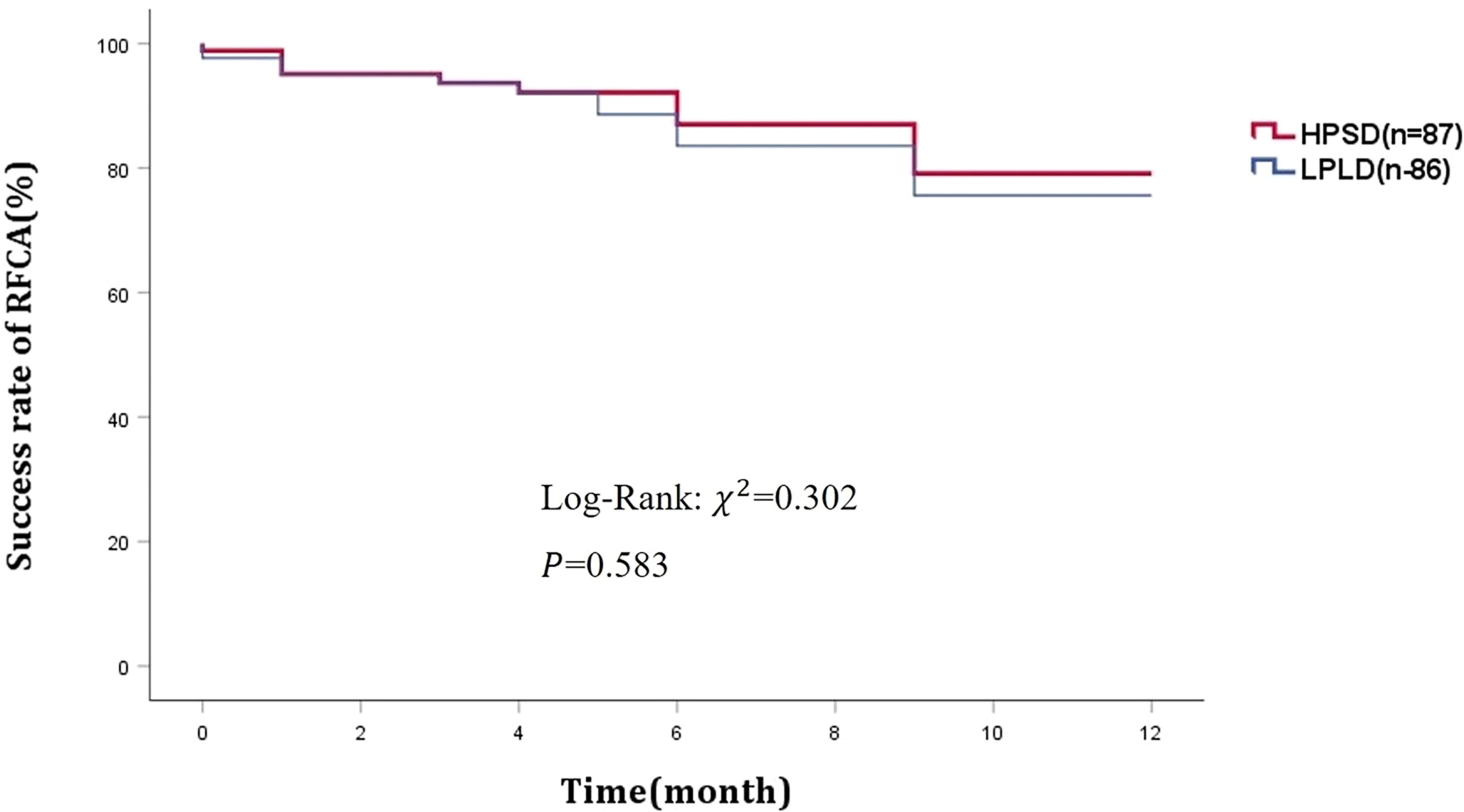
Success rate of RFCA at one-year follow-up between two groups. RFCA, radiofrequency catheter ablation.

Our procedure continues with the confirmation of the guidewire's position in the SVC using ICE. This allows for the advancement of either a ThermoCool SmartTouch Surround Flow (STSF) ablation catheter or a PENTARAY® multipolar mapping catheter through a long sheath. We then employ the EAM function to create a detailed reconstruction of the right atrium and both vena cava. Under ICE guidance, we carefully maneuver the ablation catheter to provide a precise reconstruction of the interatrial septum. To identify the optimal puncture site, we apply bending pressure and use the “tenting sign” as a visual reference. This technique ensures accurate and safe transseptal access. In preparation for the procedure, we configure a new unipolar catheter within the CARTO3 V6 system. We define the corresponding insertion points in PINBOX and establish connections using alligator clips (Figures 1-2).

In the next phase of our procedure, we advance the long sheath into the SVC. Subsequently, we insert the inner core and puncture needle [Synaptic Medical TM (Beijing) Co., Ltd.]. To enable precise tracking, we connect the proximal hub of the needle to an alligator clip, ensuring that the needle tip extends beyond the inner core of the long sheath. We leverage the CARTO3 system's newly configured unipolar electrode setting to automatically detect and display the needle tip as an electrode within the three-dimensional cardiac model. This innovative approach allows for real-time, accurate localization of the needle tip within the cardiac chamber. As we retract the needle sheath, its trajectory is clearly visible in the 3D model. We carefully align this trajectory with the pre-marked septal puncture site. The convergence of these elements confirms the precise positioning of the intended puncture point. Under ICE imaging, we observe the characteristic “tenting sign” as the puncture needle advances towards the left atrium. This visual cue, combined with our 3D mapping, guides the successful completion of the transseptal puncture (Figures 1-3).

Following successful transseptal access, we proceed with the left atrial and pulmonary vein ablation phase. For this purpose, we employ either the ablation catheter or the PENTARAY star-shaped electrode, depending on the specific requirements of the procedure. Once the initial mapping is complete, we carefully position the STSF catheter at the pulmonary vein vestibule. This strategic placement allows for precise and effective ablation of the target areas. Throughout the procedure, we continuously monitor the pericardial space using ICE imaging. This real-time visualization enables us to promptly detect any potential complications, such as pericardial effusion, ensuring patient safety throughout the intervention (Figures 1-4).

### 2.5 Radiofrequency catheter ablation

In our study, all patients were treated using the CLOSE protocol, which involved ablation index (AI)-guided point-by-point ablation (11). For 87 patients, the HPSD ablation strategy was utilized, generating lesions with 45–50 W of power for 8–15 s. In contrast, another 86 patients received LPLD ablation, using 30–35 W of power for 20–30 s (12). In our ablation protocol, we aim for a maximum inter-lesion spacing of 5 mm to ensure comprehensive coverage. We utilize Autotag parameters to configure ablation tags, setting a stability criterion of 3 mm for a 5-second ablation duration. To visually represent the efficacy of each lesion, we employ a color-coding system based on local impedance drop: white for <14 Ω, pink for 14–17 Ω, and red for ≥17 Ω. For patients presenting with *de novo* AF, we perform wide antral circumferential ablation (WACA) of the pulmonary veins (PVs). At the operator's discretion, we may implement additional lesion sets, including posterior wall isolation, anterior mitral line, and superior vena cava isolation. The contact force was generally maintained within the range of 5–15 g. High-power (45–50 W) ablation was employed, and it was observed that a contact force exceeding 20 g often led to steam pops. Conversely, maintaining a contact force below 15 g significantly reduced the occurrence of steam pops. AI values were routinely utilized during the procedure, with specific target ranges for different regions: 420–450 for the anterior wall of the right PV, 380–400 for the posterior wall, 360–380 for the left posterior wall, 430–500 for the left anterior wall, and 400–420 for the superior regions of both PVs.

The operator may also decide to create additional lines if deemed beneficial for the patient's outcome. The extra-PV linear ablations typically included the following: left atrial roof line, posterior wall BOX ablation, mitral isthmus line, and cavo-tricuspid isthmus (CTI) ablation. CTI ablation is conducted selectively, based on either the patient's history of atrial flutter or if atrial flutter manifests spontaneously during the procedure. This tailored approach allows us to address each patient's unique arrhythmia profile while maintaining a standardized framework for lesion creation and evaluation. By combining advanced mapping technologies with operator expertise, we strive to achieve optimal outcomes in our zero-fluoroscopy ablation procedures.

### 2.6 Follow-up

To evaluate the efficacy of our procedure, we conducted a comprehensive statistical analysis comparing several key metrics between the two study groups. These metrics included total procedure duration, ablation time, single-loop isolation success rate for PVs, post-procedural complications, immediate success rates, and sinus rhythm maintenance at 3, 6, and 12 months.

Following the procedure, patients were prescribed either amiodarone or propafenone for a period of 1–3 months, tailored to their individual clinical needs. Our follow-up protocol consisted of outpatient visits at 3-, 6-, and 12-months post-intervention. During these visits, we assessed patient symptoms, performed standard twelve-lead electrocardiograms (ECGs) or Holter monitoring, and conducted transthoracic echocardiography in cases of persistent atrial fibrillation with left atrial enlargement. We defined AF recurrence as either continuous AF detected on ECG after a three-month blanking period post-procedure, or episodes of atrial fibrillation/atrial tachycardia lasting ≥30 s as recorded by Holter monitoring (1).

Vascular complications are primarily defined as unilateral or bilateral femoral arteriovenous fistula, femoral artery pseudoaneurysm, femoral artery dissection, femoral vein thrombosis.

### 2.7 Statistical analyses

In our statistical analysis, we presented quantitative variables as mean values accompanied by their standard deviations, while qualitative variables were reported as absolute numbers and percentages. To compare multiple datasets, we employed the independent two-sample *t*-test, which allowed us to assess significant differences between groups. For categorical variables, we utilized either the chi-square test or Fisher's exact test, depending on the nature of the data. To evaluate the long-term success of AF ablation, we applied the Kaplan-Meier method, analyzing outcomes over the 12-month follow-up period. This approach enabled us to generate survival curves, providing a visual representation of procedure efficacy over time. Throughout our analysis, we considered a two-sided *P*-value of less than 0.05 as indicative of statistical significance. This threshold allowed us to identify meaningful differences and trends in our data. All statistical computations and analyses were conducted using IBM SPSS Statistics, version 26.0 (IBM Corporation, Armonk, New York, USA).

## 3 Results

### 3.1 Patient characteristics

Patient demographic characteristics are summarized in Table 1. There were no statistically significant differences between the two groups in terms of gender, age, atrial fibrillation type, left atrial anterior-posterior diameter, left ventricular ejection fraction, comorbidities such as hypertension, diabetes, coronary heart disease, and CHA2DS2-VASc score. In our study, the LPLD group (*n* = 86) had 42 patients with persistent AF, accounting for 48.8%, while the HPSD group (*n* = 87) had 45 patients with persistent AF, accounting for 51.7%. Each group had one patient with persistent AF who did not take oral amiodarone due to thyroid dysfunction (Table 1). Oral amiodarone was discontinued at least 7 days prior to the procedure.

**Table 1:** Patients’ demographic characteristics.

	LPLD (*n* = 86)	HPSD (*n* = 87)	*P*-value
Age (years)	66.9 ± 11.1	63.7 ± 12.1	0.076
Male, *n* (%)	45 (52.3)	55 (63.2)	0.147
Hypertension, *n* (%)	58 (67.4)	51 (58.6)	0.230
Diabetes, *n* (%)	23 (26.7)	22 (25.3)	0.827
CAD, *n* (%)	29 (33.7)	30 (34.5)	0.916
LA diameter (mm)	41.8 ± 6.4	42.7 ± 5.9	0.344
LVEF (%)	59.4 ± 8.1	61.4 ± 6.3	0.073
Persistent AF, *n* (%)	42 (48.8)	45 (51.7)	0.704
CHA_2_DS_2_-VASc Score	3.4 ± 1.5	3.0 ± 1.6	0.142
Oral amiodarone, *n* (%)	41 (47.7)	44 (50.6)	0.697

Mean values ± standard deviation, and% (n) were reported for variables, respectively. LPLD, low-power long-duration; HPSD, high-power short-duration; CAD, coronary artery disease; LA, left atrium; LVEF, left ventricular ejection fraction; AF, atrial fibrillation.

### 3.2 Procedural data

The procedural data are summarized in Table 2. All procedures were successfully completed under ICE-guided zero-fluoroscopy, establishing a feasible and reliable workflow. In terms of total procedure time, the LPLD group had an average of 130.5 ± 26.3 min, whereas the HPSD group had an average of 115.8 ± 30.8 min. For the duration of ablation, the LPLD group averaged 30.0 ± 4.1 min, while the HPSD group averaged 14.9 ± 2.3 min. The procedure and ablation times were significantly shorter in the HPSD group compared to the LPLD group. This suggests that the HPSD strategy may offer increased efficiency without compromising the effectiveness or safety of the procedure. Other metrics, including the duration of TSP, immediate success rate of PVI, success rate of PVI in a single circle, the number of ablation points, and the rate of extra-PV linear ablation, showed no statistical difference between the two groups.

**Table 2:** Patients’ procedural data.

	LPLD (*n* = 86)	HPSD (*n* = 87)	*P*-value
Duration of TSP (min)	51.7 ± 9.0	50.0 ± 10.9	0.273
Total procedure time (min)	130.5 ± 26.3	115.8 ± 30.8	0.001
Duration of ablation (min)	30.0 ± 4.1	14.9 ± 2.3	<0.001
Immediate success rate of PVI, *n* (%)	86 (100)	87 (100)	1.000
Success rate of PVI in single circle, *n* (%)	82 (95.3)	86 (98.9)	0.169
Number of ablation points	74.2 ± 14.6	71.6 ± 8.4	0.159
Extra-PV linear ablation, *n* (%)	42 (48.8)	45 (51.7)	0.704

Mean values ± standard deviation), and% (n) were reported for variables, respectively. TSP, transseptal puncture; PVI; pulmonary vein isolation.

### 3.3 Follow-up

At one-year follow-up, sinus rhythm was maintained in 77 patients in the HPSD group and 74 patients in the LPLD group, with no significant difference between the two group (Figure 2). Postoperative complications occurred in 5 patients in the HPSD group and 3 patients in the LPLD group. Two cases of small femoral vein thrombosis occurred in each group postoperatively. After treatment with low molecular weight heparin, the thrombi resolved. Follow-up examinations after discharge revealed no recurrence of femoral vein thrombosis in any of these cases. Importantly, there were no major adverse cardiac and cerebrovascular events (MACCE) in either group (Table 3).

**Table 3:** Postoperative complications and MACCE at one-year follow-up.

	LPLD (*n* = 86)	HPSD (*n* = 87)	*P*-value
Postoperative complications, *n* (%)			
Pericardial tamponade	1 (1.16)	3 (3.45)	0.621
Vascular complications, *n* (%)			
Femoral arteriovenous fistula	0 (0)	0 (0)	–
Femoral artery pseudoaneurysm	0 (0)	0 (0)	–
Femoral artery dissection	0 (0)	0 (0)	–
Femoral vein thrombosis	2 (2.33)	2 (2.30)	1.000
Esophageal fistula	0 (0)	0 (0)	–
Phrenic nerve injury	0 (0)	0 (0)	–
MACCE, *n* (%)			
Death	0 (0)	0 (0)	–
Stroke	0 (0)	0 (0)	–
Pericarditis	0 (0)	0 (0)	–
Hematoma	0 (0)	0 (0)	–
DVT/PE	0 (0)	0 (0)	–

MACCE, major adverse cardiac and cerebrovascular events; DVT, deep vein thrombosis; PE, pulmonary embolism.

## 4 Discussion

This study compares two ICE-guided zero-fluoroscopy PVI strategies for AF patients: HPSD vs. LPLD. The HPSD strategy is not inferior to the traditional LPLD strategy, demonstrating similar acute and long-term outcomes. However, the HPSD strategy offers the advantage of quicker procedures with shorter ablation times.

In recent years, there has been a growing body of clinical research on the application of HPSD strategies in catheter ablation for atrial fibrillation. Ücer E et al. (13) evaluated the effectiveness and safety of HPSD compared to traditional power settings in catheter ablation for atrial fibrillation. Their findings indicated that HPSD ablation was more likely to cause permanent damage, leading to sustained pulmonary vein bidirectional block. Similarly, studies by Do U and Shin et al. (6, 14) demonstrated that HPSD ablation offered shorter procedure times and higher efficiency while maintaining safety. Shi LB et al. (15) observed a more rapid decline in impedance and quicker disappearance of electrical potential with HPSD ablation. Baher A et al. (16) reported that HPSD ablation resulted in larger but shallower areas of tissue injury, which reduced the likelihood of steam pops (“POP”) and lowered the risk of esophageal injury. Chen et al. (17) conducted a study under Ablation Index (AI) guidance, showing that HPSD ablation was equally safe and effective, with success rates comparable to traditional power settings but with higher efficiency and shorter procedure times. Tscholl V. et al. (18), using HPSD ablation for tricuspid isthmus-dependent flutter, also confirmed its safety and reliability. Kusa S. et al. (19) demonstrated the safety and efficacy of HPSD during SVC isolation.

In summary, accumulating evidence supports that HPSD catheter ablation for atrial fibrillation is safe and reliable, with a trend toward fewer complications and improved long-term success rates. In this study, the HPSD group utilized an STSF ablation catheter with power settings of 45–50 W. The findings confirmed that HPSD ablation, applied for 10–15 s at an AI value equivalent to that of traditional power (30–35 W) ablation over 30–35 s, required less time and demonstrated greater efficiency at the same AI value. However, the incidence of steam pops appeared to be higher in the HPSD group, potentially due to the greater energy output over a shorter period, leading to faster heat accumulation. Despite this, no cases of cardiac tamponade were observed, suggesting that the depth and extent of damage caused by steam pops were limited, not penetrating the entire myocardium. In clinical practice, it has been noted that controlling the pressure at the catheter tip during HPSD ablation is crucial; a pressure range of 10–20 g appears to lower the likelihood of steam pops. Kaneshiro et al. (20) also found that during HPSD ablation, the risk of steam pops and esophageal injury significantly increased when the tip force exceeded 20 g.

The advent of 3D EAM systems has enabled interventional cardiologists to conduct ablation procedures with minimal or no radiation exposure (21). Studies have confirmed the safety of reduced or zero-fluoroscopy ablation for supraventricular arrhythmias (22, 23). A meta-analysis, including 1,593 patients, demonstrated that zero-fluoroscopy catheter ablation is not only safe and effective but also results in significantly shorter procedure times (24). These findings align with the results obtained in our study, providing further corroboration of the observed phenomena. In clinical practice, the primary challenge in achieving complete zero-fluoroscopy lies in TSP. Recent studies have demonstrated various methods to achieve zero-fluoroscopy transseptal puncture. Yu et al. (25) employed the CARTO 3D mapping system (CARTO®; Biosense Webster, Diamond Bar, CA, USA) to map the fossa ovalis and delineate its boundaries through voltage mapping, enabling precise localization of the TSP site within the fossa ovalis. However, this approach demands substantial practice, a thorough understanding of the interatrial septum anatomy, and the ability to accurately identify the fossa ovalis and its surrounding electrical potentials. In our study, we observed that in some patients, the voltage at the center of the fossa ovalis did not significantly decrease, complicating the accurate localization of the puncture site using this method. Žižek et al. (26) explored the use of ICE to guide TSP by visualizing the needle's trajectory and navigating it through the fossa ovalis under ultrasound imaging. The “tenting sign” visible on ICE confirmed successful transseptal puncture. Tahin T et al. (27) further simplified the TSP procedure by integrating ICE with wire localization of the fossa ovalis, accumulating practical expertise. While ICE-guided TSP methods are now widely adopted, ICE imaging is essentially a two-dimensional ultrasound technique, making it challenging to monitor the entire trajectory of the puncture needle from the SVC to the fossa ovalis. This often necessitates multiple adjustments to the ultrasound probe during the procedure.

The integration of ICE in AF ablation procedures has seen significant advancements, particularly with the development of 3D real-time ICE. Recent studies have highlighted the benefits of using 3D ICE for improved anatomical visualization, procedural precision, and safety. The use of ICE, especially 3D real-time ICE, enables operators to effectively guide transseptal punctures, monitor catheter positioning, and assess lesion formation (28). This real-time imaging approach, combined with EAM, has paved the way for safer, zero-fluoroscopy procedures, minimizing radiation risks for both patients and medical staff. Additionally, the improved spatial resolution and depth perception offered by 3D ICE help in precise catheter manipulation, leading to potentially higher procedural success rates and reduced complication risks (29). These innovations underscore the growing importance of ICE in modern electrophysiology, making it a valuable tool in the shift towards more efficient and radiation-free AF ablation strategies.

In recent years, the adoption of visualizable steerable sheaths, such as Vizigo, has provided significant advancements in reducing fluoroscopy times during PVI procedures for AF. Visualizable sheaths, which can be tracked using EAMS, enhance procedural efficiency and safety by minimizing reliance on fluoroscopy (30). Randomized and observational studies have demonstrated that these sheaths facilitate catheter positioning and mapping without fluoroscopic guidance, thereby reducing radiation exposure for both patients and medical personnel. For instance, a randomized trial comparing visualizable and standard, non-visualizable steerable sheaths showed substantial reductions in fluoroscopy usage with the Vizigo sheath, while an observational study highlighted its positive impact on the procedural workflow and fluoroscopy time. Including visualizable sheaths like Vizigo in AF ablation workflows aligns with the ALARA (As Low As Reasonably Achievable) principle, making it a preferred choice in modern PVI techniques (31).

The significant innovation in our study is the combination of ICE with EAM, allowing real-time visualization of the catheter and needle tip throughout its trajectory on a three-dimensional model. Once the needle tip reaches the intended puncture site under direct ICE visualization, there is no need to adjust or reposition the ICE catheter, thereby simplifying the procedure and reducing the number of operational steps. Given that the ablation catheter marker points typically have a diameter of 4 mm, combining ICE with EAM guidance allows for a precision of within 4 mm during TSP procedures. In our study, dual confirmation from both ICE and EAM techniques enabled accurate completion of TSP in all 173 cases, with no related complications observed. This approach proved particularly effective in challenging situations, such as cardiac rotation or an enlarged right atrium with interatrial septal aneurysm formation, ensuring precise and safe execution of the TSP procedures.

### 4.1 Limitations

The present study has some limitations. First, the study included a relatively small number of patients, which may limit the generalizability of the findings to a broader population. Second, the study was conducted at a single center and employed a retrospective design, which could introduce referral bias and limit the external validity of the results. Third, although the study demonstrates the feasibility and safety of HPSD ablation under ICE guidance, the overall clinical evidence supporting this technique remains limited, especially in comparison to more established methods. Forth, the success of combining ICE with EAM for zero-fluoroscopy procedures heavily depends on the operator's experience and expertise, which may not be consistent across different clinical settings. Finally, the study's follow-up period was relatively short, restricting the ability to assess the long-term efficacy and safety of the HPSD strategy.

## 5 Conclusion

A zero-fluoroscopy workflow utilizing an EAM system combined with ICE appears to be both feasible and safe for ablation in AF patients. In patients undergoing ICE-guided zero-fluoroscopy ablation, the HPSD strategy is comparable to LPLD ablation in effectiveness while offering the benefit of shorter procedure and ablation times.

## Data Availability

The raw data supporting the conclusions of this article will be made available by the authors, without undue reservation.
